# Associations Between Altered Cerebral Activity Patterns and Psychosocial Disorders in Patients With Psychogenic Erectile Dysfunction: A Mediation Analysis of fMRI

**DOI:** 10.3389/fpsyt.2020.583619

**Published:** 2020-10-27

**Authors:** Tao Yin, Qi Liu, Ziyang Ma, Zhengjie Li, Ruirui Sun, Feiqiang Ren, Guangsen Li, Xiaopeng Huang, Degui Chang, Peihai Zhang

**Affiliations:** ^1^Acupuncture and Tuina School/The 3rd Teaching Hospital, Chengdu University of Traditional Chinese Medicine, Chengdu, China; ^2^Acupuncture-Brain Science Research Center, Chengdu University of Traditional Chinese Medicine, Chengdu, China; ^3^Acupuncture and Tuina School, Shaanxi University of Chinese Medicine, Xian, China; ^4^Hospital of Chengdu University of Traditional Chinese Medicine, Chengdu, China; ^5^Clinical Medical School, Chengdu University of Traditional Chinese Medicine, Chengdu, China

**Keywords:** psychogenic erectile dysfunction, psychosocial status, amplitude of low-frequency fluctuations, functional connectivity, fMRI, mediation analysis

## Abstract

Previous studies had illustrated the significant neural pathological changes in patients with psychogenic erectile dysfunction (pED), while few works focused on the neural underpinning of the psychosocial status in patients with pED. This study aimed to investigate the associations among the altered cerebral activity patterns, impaired erectile function, and the disrupted psychosocial status in patients with pED. Thirty-two patients with pED and 28 healthy controls (HCs) were included. The amplitude of low-frequency fluctuations (ALFF), region-of-interest-based functional connectivity (FC), as well as *Pearson* correlation analyses and mediation analyses between neuroimaging outcomes and clinical outcomes were performed. Compared to HCs, patients with pED manifested lower erectile function, disrupted psychosocial status, as well as decreased ALFF in the left dorsolateral prefrontal cortex (dlPFC) and reduced FC between the left dlPFC and left angular gyrus, and left posterior cingulate cortex (PCC) and precuneus, which belonged to the default mode network (DMN). Moreover, both the ALFF of the left dlPFC and FC between the left dlPFC and left PCC and precuneus were significantly correlated with the sexual function and psychosocial status in patients with pED. The disrupted psychosocial status mediated the influence of atypical FC between dlPFC and DMN on decreased erectile function. This study widened our understanding of the important role of psychosocial disorders in pathological neural changes in patients with pED.

## Introduction

Erectile dysfunction (ED), the most common male sexual disorder, is defined as the persistent inability to attain or maintain sufficient penile erection for successful sexual performance (1). Population-based cross-sectional studies estimated that 11% of men in their 30s and 24% of men in their 40s suffered from this sexual dysfunction (2, 3). A recent review on the epidemiological surveys of ED similarly indicated that the prevalence of ED among young people was as high as 30% (4). ED is now recognized as a major public health issue, not only for its high prevalence but also for its severe impacts on sexual satisfaction and quality of life (QOL) of patients and their partners (5–8). Among the different subtypes of ED, psychogenic ED (pED) has always been a focus of researchers for the high incidence and complex pathogenesis (7, 9, 10). Different from organic ED, which has physical causes and evident pathological characteristics, pED is generally caused by psychosocial factors and lacks specific biomarkers (11, 12).

Male sexual arousal is described as a complicated biopsychosocial process that involves the coordination of psychological, neurological, endocrine, and vascular systems (13). With the application of neuroimaging technologies, the close correlation between sexuality and the central nervous system (CNS) is becoming clearer (14–16). Many male sexual dysfunctions, such as ED (17–22), premature ejaculation (23, 24), and anejaculation (25) have been detected to be associated with alterations in the structure and function of the brain. For example, patients with pED demonstrated the atrophied gray matter volume (17, 19), altered white matter microstructure (26), aberrant activity patterns (20), and disrupted topological properties (22) in multiple brain regions, such as prefrontal cortex, cingulate cortex, and insular cortex. These studies indicated that the structural and functional aberrancies of the brain might be the critical pathogenesis properties of pED. However, most of these previous studies paid primary attention to the neural mechanism of the low erectile function, and few works focused on the associations between the altered cerebral activity and the psychosocial status of pED patients. As a typical psychosocial disorder, patients with pED demonstrated not only decreased erectile function but also severe symptom-related psychosocial disorders, such as poor sexual relations, diminished sexual satisfaction, and low self-esteem. Therefore, in addition to investigating the correlations between the altered cerebral activity properties and the physiological function, it is necessary to explore the neural underpinning of the disrupted psychosocial status in patients with pED, so that to more fully understand the neuropathological features underlying pED. The amplitude of low-frequency fluctuations (ALFF) and region-of-interest (ROI)-based functional connectivity (FC) are two commonly used approaches to investigate human brain activity patterns. The ALFF is a data-driven algorithm to measure the spontaneous activity of the brain (27), and the ROI-based FC is a purpose-driven algorithm that reflects the synchronization of different brain regions and indicates the tendency of cortical networks to be co-activated (28). The combination of ALFF and ROI-based FC provides valuable perspectives for detecting the neural underpinning of diseases (29, 30).

In this study, we aimed to investigate the alterations of spontaneous cerebral activity and synchronous cerebral activity of patients with pED using ALFF and ROI-based FC analysis methods, and to examine the potential associations among the altered cerebral activity patterns and the decreased erectile function as well as the disrupted psychosocial status in patients with pED. To deepen the understanding of the impacts of psychosocial status on clinical symptoms and cerebral activity patterns, the mediation analysis with psychological status as a mediator was also conducted. We hypothesized that the cerebral activity patterns were altered in patients with pED compared to healthy controls (HCs), and that the disordered psychosocial status mediated the relationship between the aberrant cerebral activity patterns and the low erectile function.

## Materials and Methods

### Participants Selection

Thirty-two patients with pED and 28 HCs were enrolled in this study. The patients were recruited at the outpatient department of Andrology in the Hospital of Chengdu University of Traditional Chinese Medicine and Sichuan Integrative Medicine Hospital from November 2018 to April 2019. All the potential patients were diagnosed with comprehensive history taking, physical examinations, laboratory tests, and specific examinations. The details of the preceding items could be found in our early study (26). Patients were included if they fulfilled the following inclusion criteria: (1) matched the diagnosis criteria of the guidelines for the diagnosis and treatment of Chinese male diseases (2013 edition) (31); (2) were right-handed and 20 to 45 years old; (3) had impotence symptoms for at least 6 months; (4) had a stable heterosexual partner for more than 1 year; (5) avoided taking medications affecting sexual function over 30 days before enrollment; and (6) signed the informed consent. Patients were excluded if they: (1) were diagnosed with organic ED or mixed ED by history taking or examinations; (2) suffered from any organic or metabolic disease of urological, cardiovascular, respiratory, gastrointestinal system or had other severe primary diseases; (3) were alcohol or drug addicts or had neuropsychiatric disorders; (4) had a history of head trauma or urological surgery; (5) were participating in other current clinical trials; or (6) had any contraindication of MRI scans, such as implanted ferromagnetic metal and claustrophobia. The 28 right-handed HCs (ranged from 20 to 45 years old) were recruited by advertisement. These volunteers had never been diagnosed with ED or other sexual dysfunction and undergone the same clinical examinations as patients before inclusion. HCs with a history of neurological or psychiatric disorders, or urinary tract surgery were excluded.

### Symptom Assessments

In addition to a brief assessment of sexual function using the International Index of Erectile Function 5 (IIEF-5) routinely, we selected the Self-Esteem and Relationship Questionnaire (SEARQ) and Quality of Erection Questionnaire (QEQ) to assess each participant's psychosocial status (self-esteem, sexual relationships, and erectile satisfaction). Moreover, the Self-Rating Anxiety Scale (SAS) (32) and the Self-Rating Depression Scale (SDS) (33) were also exploited to measure the mental states. The IIEF-5 is a multidimensional self-reported instrument for the assessment of male sexual function (34). It is recommended as the standard screening and diagnostic tool for ED in clinical (35). The 14-item SEARQ is a commonly used patient-administered and disease-specific psychosocial questionnaire to evaluate the psychosocial status of males. It contains three dimensions: self-esteem, sexual confidence, and sexual relationship (36–38). The QEQ is a self-reported questionnaire that specifically evaluates male satisfaction with erectile quality (39). The symptoms assessments were performed prior to the MRI scans, and the lower scores of these questionnaires indicated more severe symptoms, lower self-esteem, poorer sexual relationships, and lower erectile satisfaction.

### MRI Data Acquisition

The MRI data were acquired with a 3.0T GE scanner at the Department of Radiology, Hospital of Chengdu University of Traditional Chinese Medicine, Chengdu, China. Each participant underwent a high-resolution three-dimensional T1-weighted imaging scan and a resting-state blood oxygenation level-dependent fMRI (BOLD-fMRI) sequence scan with eyes blindfolded and ears plugged. The structural scanning parameters were as follows: repetition time /echo time= 1,900/2.26 ms, slices = 176, slice thickness = 1 mm, matrix size = 256 × 256, field of view = 256 × 256 mm^2^. The BOLD-fMRI data were acquired with the following parameters: repetition time/echo time = 2,000/30 ms, slices = 30, slice thickness = 5 mm, flip angle = 90°, matrix size = 64 × 64, field of view = 240 × 240 mm^2^, total volume = 180.

### Data Analysis

#### Clinical Data Analysis

The demographic characteristics and clinical measurements were analyzed *via* SPSS 20.0 (SPSS Inc. USA). Age, Body Mass Index (BMI), IIEF-5 score, SEARQ score, and QEQ score were described as mean ± standard deviation and compared by the two-sample *t*-test. The significance threshold was set at 0.05 (two-tailed).

#### MRI Data Analysis

The MRI data were preprocessed and analyzed by Statistical Parametric Mapping 12.0 (SPM12, http://www.fil.ion.ucl.ac.uk/spm), Data Processing Assistant for Resting-State fMRI (DPARSF) (40) (http://rfmri.org/DPARSF), and FC toolbox (CONN) (http://www.nitrc.org/projects/conn) working on MATLAB 2013b (Mathworks Inc. USA).

#### MRI Data Preprocessing

The data preprocessing were carried out with DPARSF, including (1) discarded the first 10 timepoints; (2) perfomed slice-timing correction; (3) performed realignment and discarded the subjects with a mean framewise displacement (FD) value exceeding 0.2 mm or a maximum displacement greater than one voxel size (41, 42); (4) reoriented the functional images and T1 images with six rigid-body parameters; (5) coregistered the T1 images to functional space for each subject, respectively, and performed new segment and DARTEL (diffeomorphic anatomical registration through exponentiated lie algebra); (6) regressed out nuisance covariates including linear trend, white matter and cerebrospinal fluid signals, and head motion parameters [Friston 24 parameter model (43, 44)]; (7) normalized the functional images to MNI space and then resampled the functional images to 3 mm cubic voxels; (8) smoothed images with a 6 mm Gaussian kernel of full-width at half maximum; and (9) performed temporal filtering (0.01–0.08 Hz) (45). Following Yan et al.'s recommendation (44), the scrubbing procedure was conducted to remove the bad time points when performing FC analysis. The bad time points were defined as those whose FD was larger than 0.5 mm.

#### ALFF Analysis

The zALFF maps, which normalized with the normal z-transformation, were applied to examine the differences of the spontaneous cerebral activity between patients with pED and HCs. The ALFF analysis was conducted with a two-sample *t*-test *via* SPM12. The multiple-comparisons corrections were performed based on Gaussian random field theory (46, 47) and the threshold was set to voxel-level *p* < 0.001 uncorrected and cluster-level *p* < 0.05 familywise error (FWE) corrected (48).

#### ROI-Based FC Analysis

The voxels exceeding the established statistical threshold in the ALFF analysis were selected as the ROI, and the ROI-based FC analysis was performed with CONN. We first extracted the average BOLD time-series of the ROI and calculated the temporal synchronization between ROI and other voxels across the whole brain. And then, transferred the correlation coefficients into z-scores using Fisher's transformation to allow for the normal distribution. Again, the two-sample *t*-test with the same statistical threshold as ALFF analysis was performed to investigate the ROI-based FC performance of patients with pED and HCs. Both age and BMI were controlled at ALFF and ROI-based FC analysis.

#### Correlation Analysis

To investigate the associations between symptom severity, psychosocial status, and brain functional alterations in pED, the clusterwise correlation analyses were performed between the ALFF/FC value and duration/ IIEF-5 score/ SEARQ score/ QEQ score in pED group, with age, BMI, and mean FD controlled. The significance threshold was set to *p* < 0.05 with Bonferroni corrected.

#### Mediation Analysis

Mediation analyses were conducted within SPSS 20.0 using the PROCESS macro (49). A full mediation model (model 4) was applied to examine the potential mediating role of the psychosocial status (lower SEARQ score) on the relationship between cerebral activity patterns and decreased erectile function (lower IIEF-5 score), with age, BMI, and mean FD included as covariates. Similar to the previous studies (50, 51), the non-parametric bootstrapping analysis was utilized to calculate the desired statistic in each resample. Bias-corrected bootstrap confidence intervals (CI) were estimated from 10,000 bootstrap samples, and the mediation effects were considered statistically significant if the bootstrapped 95% CI did not include zero.

## Results

All the participants completed the MRI scan. Two HCs were excluded due to incomplete functional images. One patient and two HCs were excluded due to excessive head motion. Therefore, 31 patients with pED and 24 HCs were included in the final data analysis. There was no significant difference in mean FD between patients and HCs (*p* > 0.05).

### Demographic and Clinical Characteristics

There was no significant difference in age, BMI, SAS, and SDS between patients with pED and HCs (*p* > 0.05). While patients with pED had lower IIEF-5 score, SEARQ score, and QEQ score than HCs (*p* < 0.001), which indicated the impaired erectile function, damaged sexual relationships, decreased self-esteem, and diminished erectile satisfaction in pED group (Table 1). In addition, The IIEF-5 was found to be positively correlated with the SEAR score (*r* = 0.688, *p* < 0.0001) and QEQ score (*r* = 0.754, *p* < 0.0001), but not with the duration, SAS, or SDS in patients with pED.

**Table 1:** The comparisons of demographic and clinical characteristics between patients with pED and HCs.

	**Age (Year)**	**BMI**	**Duration (Months)**	**IIEF-5**	**SEARQ**	**QEQ**	**SAS**	**SDS**	**Mean FD**
pED (*n* =31)	33.16 ± 5.89	22.16 ± 1.87	33.06 ± 28.67	13.97 ± 3.6	37.58 ± 7.96	35.48 ± 15.72	35.24 ± 7.92	35.97 ± 8.02	0.122 ± 0.037
HCs (*n* =24)	31.17 ± 6.57	22.7 ± 3.54	/	22.21 ± 0.98	61.92 ± 3.73	81.36 ± 6.8	34.01 ± 5.33	33.49 ± 5.26	0.113 ± 0.451
*p*-value	0.241	0.467	/	<0.001	<0.001	<0.001	0.515	0.196	0.385

*No significant difference was obtained in age, BMI, SAS, SDS, and mean FD (p > 0.05). Patients with pED had lower IIEF-5 score, SEARQ score, and QEQ score than HCs (p < 0.001). pED, psychogenic erectile dysfunction; HCs, healthy controls; BMI, Body Mass Index; IIEF-5, International Index of Erectile Function 5; SEARQ, Self-esteem and Relationship Questionnaire; QEQ, Quality of Erection Questionnaire; SAS, Self-rating Anxiety Scale; SDS, Self-rating Depression Scale; FD, framewise displacement*.

### Between-Group ALFF Comparisons

**Figure 1:**
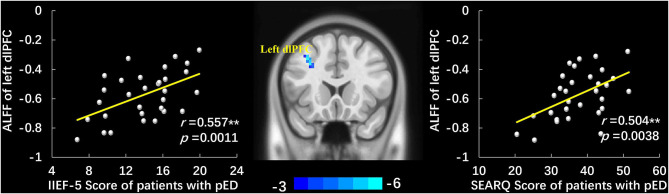
The group-differences of ALFF between patients with pED and HCs and the scatter plots of correlation analyses. Patients with pED demonstrated lower ALFF in left dlPFC than HCs (voxel-level *p* < 0.001 uncorrected, cluster-level *p* < 0.05 FWE corrected, cluster size > 20 voxels). ALFF value of the left dlPFC was positively correlated with IIEF-5 score and SEARQ score after adjusting age, BMI, and mean FD in pED group (*p* < 0.05, Bonferroni corrected, **). ALFF, amplitude of low-frequency fluctuations; pED, psychogenic erectile dysfunction; dlPFC, dorsolateral prefrontal cortex; IIEF-5, International Index of Erectile Function 5; SEARQ, Self-esteem and Relationship Questionnaire.

Compared to HCs, patients with pED demonstrated significant ALLF decrease at the left dlPFC (voxel-level *p* < 0.001 uncorrected, cluster-level *p* < 0.05 FWE corrected, cluster size > 20 voxels) (Figure 1, Table 2). No region with higher ALFF was observed in pED group. The correlation analysis demonstrated that the ALFF value of the left dlPFC was positively correlated with the IIEF-5 score (*r* = 0.557, *p* = 0.0011) and SEARQ score (*r* = 0.504, *p* = 0.0038), while not with duration and QEQ scores in patients with pED after controlling age, BMI, and mean FD (Bonferroni correction, *p* < 0.05/4 = 0.0125) (Figure 1).

**Table 2:** Differences in ALFF between patients with pED and HCs.

**Contrast**	**Foci**	**Voxels**	**BA**	**MNI coordinate (x, y, z)**			***t-*value**
pED < HCs	L dlPFC	22	46	−33 −33	18 24	42 51	−5.24 −3.44

*Patients with pED manifested lower ALFF in left dlPFC than HCs (voxel-level p < 0.001 uncorrected, cluster-level p < 0.05 FWE corrected, cluster size > 20 voxels). pED, psychogenic erectile dysfunction; HCs, healthy controls; L, left; dlPFC, dorsolateral prefrontal cortex; BA, Brodmann area; MNI, Montreal Neurological Institute*.

### Between-Group FC Comparisons

**Figure 2:**
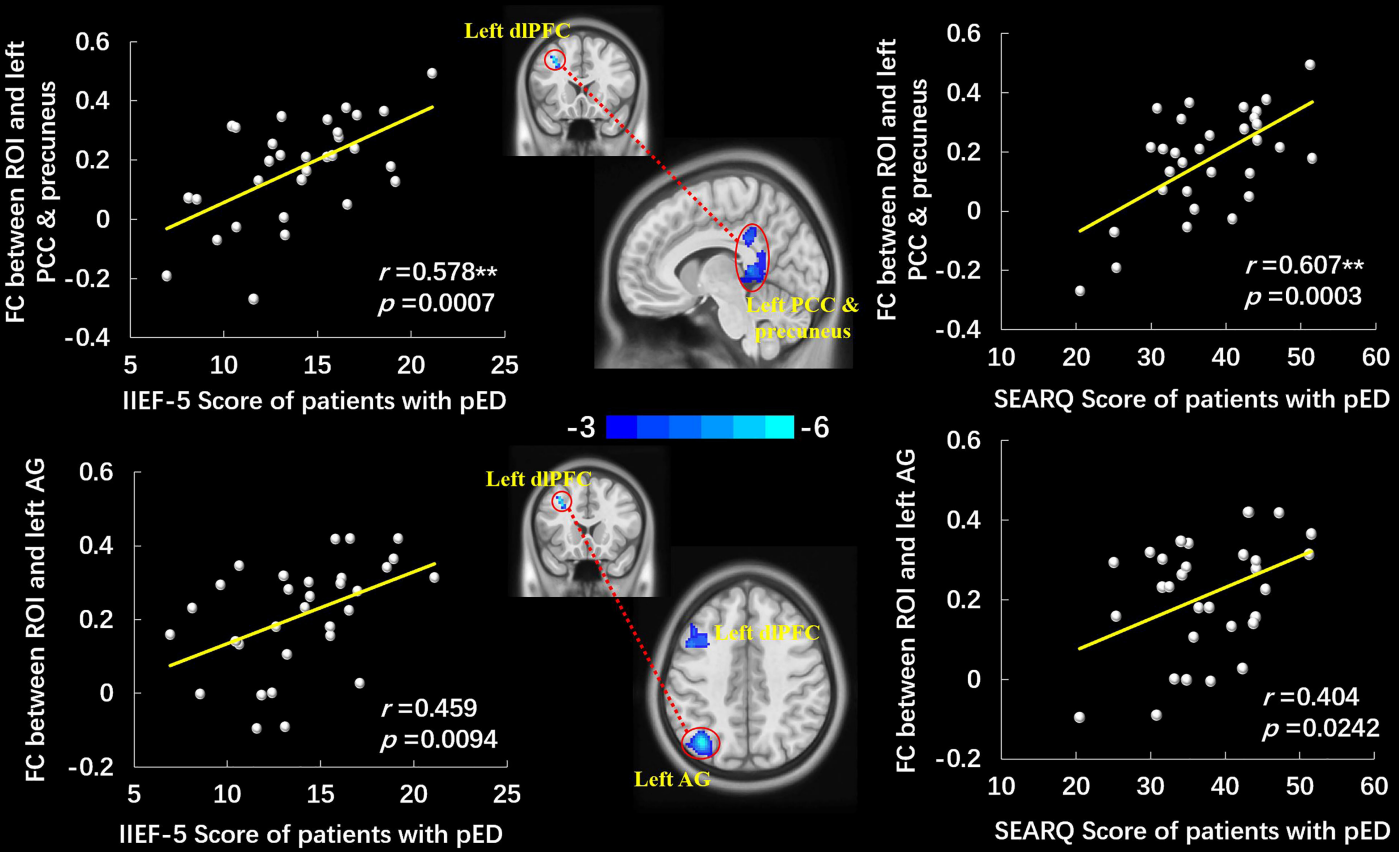
The group-differences of left dlPFC-based FC between patients with pED and HCs and the scatter plots of correlation analyses. Patients with pED demonstrated decreased FC between the left dlPFC and left PCC and precuneus, left AG, and left dlPFC (voxel-level *p* < 0.001 uncorrected, cluster-level *p* < 0.05 FWE corrected, cluster size > 20 voxels). FC between the left dlPFC and left PCC and precuneus was positively correlated with IIEF-5 score and SEARQ score (*p* < 0.05, Bonferroni corrected, **), and FC between the left dlPFC and left AG was positively correlated with IIEF-5 score and SEARQ score (*p* < 0.05, uncorrected) after adjusting age, BMI, and mean FD in pED group. FC, functional connectivity; dlPFC, dorsolateral prefrontal cortex; AG, angular gyrus; PCC, posterior cingulate cortex; IIEF-5, International Index of Erectile Function 5; SEARQ, Self-esteem and Relationship Questionnaire.

The left dlPFC, which manifested significantly lower ALFF in pED group, was selected as the ROI. Compared to HCs, the decreased FC were observed between the ROI and the left dlPFC, left angular gyrus (AG), and left PCC and precuneus in patients with pED (voxel-level *p* < 0.001 uncorrected, cluster-level *p* < 0.05 FWE corrected, cluster size > 20 voxels) (Figure 2, Table 3). No significantly increased FC between ROI and voxels was found in pED group. The correlation analysis manifested that the FC between ROI and left PCC and precuneus was positively correlated with IIEF-5 score (*r* = 0.578, *p* = 0.0007) and SEARQ score (*r* = 0.607, *p* = 0.0003) in patients with pED after adjusting the impacts of age, BMI, and mean FD (Bonferroni correction, *p* < 0.05/12 = 0.0042). Furthermore, FC between ROI and left AG was found to be associated with the IIEF-5 score (*r* = 0.459, *p* = 0.0094) and SEARQ score (*r* = 0.404, *p* = 0.0242) in patients with pED at the threshold of uncorrected *p* < 0.05. However, the above correlation results were no longer significant after Bonferroni correction (Figure 2).

**Table 3:** Differences of ROI-based FC between patients with pED and HCs.

**Contrast**	**Foci**	**Voxels**	**BA**	**MNI coordinate (x, y, z)**			***t*-value**
	L dlPFC	581	46	−34	18	40	−4.15
	L AG	552	39	−38	−68	42	−5.55
pED < HCs	L PCC	539	23/29/31	−8	−40	36	−3.82
	L precuneus		31	−2	−64	28	−3.77

*Patients with pED manifested lower FC between ROI and left dlPFC, left AG, and left PCC and precuneus than HCs (voxel-level p < 0.001 uncorrected, cluster-level p < 0.05 FWE corrected, cluster size > 20 voxels). pED, psychogenic erectile dysfunction; HCs, healthy controls; L, left; dlPFC, dorsolateral prefrontal cortex; AG, angular gyrus; PCC, posterior cingulate cortex; BA, Brodmann area; MNI, Montreal Neurological Institute*.

Given the significant correlation between the duration and the SEARQ score (*r* = −0.513, *p* = 0.003), we conducted the correlation analyses between neuroimaging data and clinical symptoms with duration as an additional covariate to exclude its impact. These results are displayed at Supplementary Material.

### Mediation Analysis

**Figure 3:**
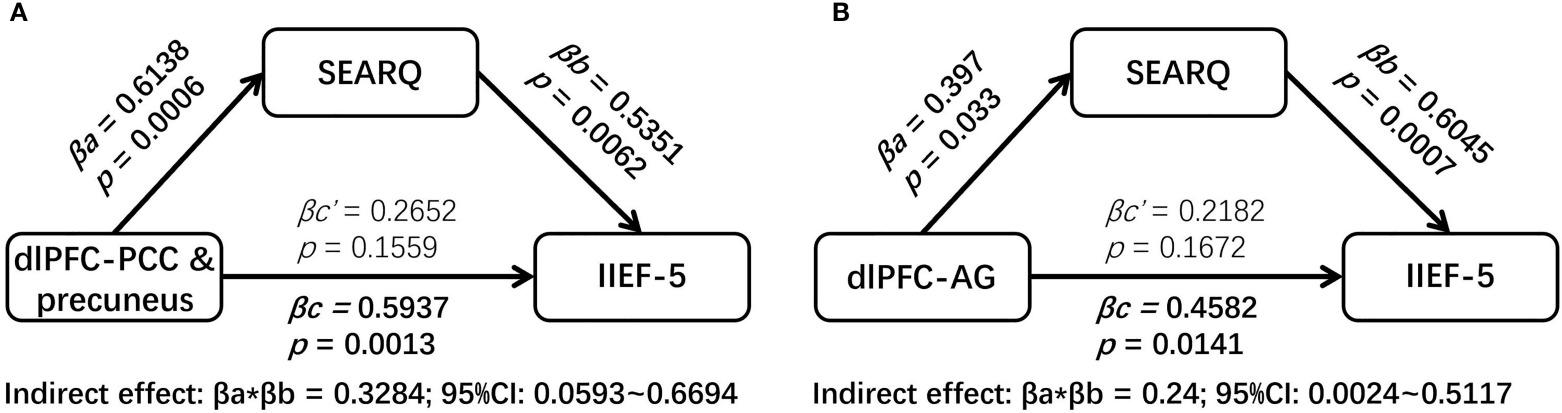
The results of mediation analyses. The SEARQ score fully mediated the impacts of left dlPFC-left PCC and precuneus connectivity **(A)** and left dlPFC-left AG connectivity **(B)** on IIEF-5 score. dlPFC, dorsolateral prefrontal cortex; PCC, posterior cingulate cortex; AG, angular gyrus; IIEF-5, International Index of Erectile Function 5; SEARQ, Self-esteem and Relationship Questionnaire.

The mediation analyses demonstrated that the associations between the altered brain activity synchronization and ED symptoms were mediated through the psychosocial status after controlling the impacts of age, BMI, and mean FD. Namely, the SEARQ score mediated the influences of the dlPFC-left PCC and precuneus connectivity on the IIEF-5 score (indirect effect = 0.3284; 95% CI: 0.0593–0.6694) (Figure 3A). Interestingly, the total effect of left dlPFC-left PCC and precuneus connectivity on IIEF-5 score was significant (βc = 0.5937, *p* = 0.0013), but after taking the significant mediation effect of SEARQ score into consideration, the remaining direct effect of cerebral activity on erectile function was reduced and no longer significant (βc‘ = 0.2652, *p* = 0.1559). Similarly, we also found the fully mediating effect of SEARQ score on the relationships between left dlPFC-left AG connectivity and IIEF-5 score (indirect effect = 0.24; 95% CI: 0.0024–0.5117) (Figure 3B). No significant mediation effect was found between ALFF value and clinical measurements.

## Discussion

This study investigated the associations among the cerebral activity patterns, erectile function, and psychosocial status in patients with pED. The current findings indicated that patients with pED manifested not only the impaired erectile function and disrupted psychosocial status, but also the lower spontaneous activity in the left dlPFC and lower synchronous activity between the left dlPFC and the left AG, left PCC and precuneus, and left dlPFC compared to HCs. Moreover, we also found that both the ALFF of left dlPFC and FC between left dlPFC and left PCC and precuneus were positively correlated with the sexual function and psychosocial status of patients with pED, and that the disrupted psychosocial status fully mediated the influence of altered brain activity synchronization on impaired erectile function.

A great number of studies have indicated that the abnormal psychosocial status was the important cause of pED, and that patients with pED manifested significantly decreased self-esteem, damaged sexual relationships, and diminished sexual confidence than healthy men (52–54). The current findings illustrated that patients with pED displayed symptom-related SEARQ scores and QEQ scores decrease, which was consistent with previous studies (54–56). They all indicated that patients with pED had significant psychosocial disorders that were closely related to the severity of symptoms. Another interesting finding of the present study was that the psychological status fully mediated the influences of atypical FC on the clinical symptoms. Namely, the altered left dlPFC-left PCC and precuneus connectivity as well as left dlPFC-left AG connectivity could indirectly affect the clinical symptoms of pED patients by influencing their psychological status. This contributed to the understanding of the mechanisms behind the correlations between abnormal brain functional synchronization and impaired erectile function, and further enhanced our knowledge of the critical roles played by psychosocial disorders in the pathogenesis of pED. To some extent, this finding implied the rationale and feasibility of modulating abnormal brain activity and regulating abnormal psychosocial status to improve clinical symptoms of pED, which has been tentatively demonstrated in several clinical studies (57–59).

This result showed that patients with pED had reduced spontaneous activity at the left dlPFC, and another study also observed the aberrant activity patterns at dlPFC in patients with pED (20). These two studies illustrated that the abnormal functional activity in the dlPFC might be an important neural pathological feature of pED. That dlPFC playing a critical role in male sexual arousal and sexual behavior has been extensively investigated in both healthy subjects and patients (60–62). For example, researchers demonstrated that visual sexual stimulus could significantly produce dlPFC activation and that healthy males manifested higher and more rapid dlPFC activation than females (63). Compared to HCs, patients with problematic hypersexual behavior experienced stronger sexual desire and higher dlPFC activation when exposed to the visual sexual stimulus (64). In contrast, patients with hypoactive sexual desire disorder showed lower BOLD signal activity in dlPFC during a similar stimulus (65). As the core region of central executive network (CEN) (66), dlPFC primarily implicates in response inhibition, cognitive control, and attention (62). It was reported that patients with minor dlPFC damage demonstrated disinterest in their surroundings and inhibition in behavior (67). Sexual inhibition, as one of the most critical characteristics of pED, has been found to be associated with the dlPFC activity in the previous researches (62, 68). In the current study, we found the abnormally decreased activity at left dlPFC in patients with pED, and the activity intensity of left dlPFC was positively correlated with the sexual ability and sexual satisfaction. This result identified that patients with pED had dlPFC-involved aberrant sexual inhibition and insufficient cognition and attention to sexual targets.

In addition to the altered spontaneous activity of the brain, we also detected the decreased FC between the left dlPFC and multiple key regions of default mode network (DMN) in pED group, and the FC values of ROI to foci in DMN were positively associated with the erectile function and psychosocial status. These results suggested that the abnormal synchronization of functional activity between dlPFC and regions of DMN might contribute to the pathogenesis of pED. DMN is a high-interconnected anatomical brain network that contains four functional hubs: the PCC, the precuneus, the AG, and the medial PFC (69, 70). DMN participates in regulating the male erection (62, 71–73). The aberrances of some DMN regions contribute to the abnormal sexual arousal and behavior patterns, manifesting as pED (17, 19) and other sexual dysfunctions (74, 75). DMN involves in many functions, such as self-referential introspection, self-esteem, thinking about others, remembering the past, and looking forward to the future (76–78). As the core regions of DMN, the PCC and precuneus mainly participate in autobiographical and emotional memories, as well as the integration of self-evaluation, perception, memories, and attention (69, 79, 80); while the AG engages in the manipulation of mental representations and conceptual knowledge, and helps with the recall of episodic memories (81, 82). The erection is a complex physiological process involving perception, introspection, cognitive, and affective components (83, 84). The occurrence of pED is primarily related to traumatic memories, as well as self-cognitive, affective, and interpersonal factors (9, 85). Therefore, in addition to the dlPFC-involved abnormal sexual inhibition, it seemed that patients with pED might also have DMN-related improper introspection, damaged self-esteem, overthinking about nervous sexual relationships, and undue consolidation and extraction of traumatic memories about failed intercourses. In addition, the aberrant function of DMN has been confirmed to be relevant to psychosocial stress (86), major depression (87), posttraumatic stress disorder (PTSD) (88), and many other psychosocial disorders (89, 90). As a typical biopsychosocial illness, the incidence of pED was inseparable from the influence of psychosocial factors, such as performance anxiety, nervous sexual relationships, and failed intercourse memories. Our present study found that the disordered psychosocial status mediated the impacts of dlPFC-DMN FC on impotence symptoms, which further illustrated the close associations among the aberrant psychosocial status, impaired erectile function, and altered cerebral activity patterns in patients with pED.

To some degree, the current results appeared to be able to deduce the viewpoint that patients with pED had aberrant connectivity patterns between CEN and DMN. There are complex connectivity and mutual interactions between CEN and DMN (91). The DMN is responsible for the processing of endogenous or self-referential mental activity, and the CEN participates in the processing of exogenous or cognitively demanding mental activity (92). CEN and DMN together involve in cognitive control, self-emotion regulation, and attention to the external and internal worlds. In our study, patients with pED not only demonstrated the decreased spontaneous activity of the CEN hub (dlPFC), but also manifested the reduced synchronous activity between the core regions of CEN and DMN. It indicated that patients with pED had different CEN-DMN connectivity models from healthy subjects. Similarly, the weak CEN-DMN connectivity was also detected at many other psychosocial disorders, such as major depression (93), PTSD (94), and acute stress (95). Therefore, based on the previous findings and our current results, we put forward the deduction that patients with pED had decreased synchronous activity between the CEN and the DMN.

Some limitations should be concerned in this study. First, the sample size was relatively small; studies with larger samples are encouraged to repeat the current findings. Second, because no significant difference was found between patients with pED and HCs in SAS and SDS scores, this study did not further investigate the correlation or mediation effects of imaging data and emotional condition. Third, patients enrolled in this study were aged from 20 to 45 years old, so the findings reflected the disease characteristics of younger patients only. As we know, the incidence of ED increases significantly with age, and the majority of cases are in the middle-aged and elderly population. Therefore, future studies should pay more attention to older pED patients.

## Conclusion

In conclusion, this study demonstrated that patients with pED had symptom-related abnormal psychosocial status, decreased spontaneous activity in dlPFC, and damaged FC between dlPFC and DMN, and that the disrupted psychosocial status fully mediated the influence of aberrant dlPFC-DMN connectivity on ED symptoms. This study widened our understanding of the important role of psychosocial disorders in pathological neural changes in patients with pED. The future intervention for pED should fully consider the impacts of psychosocial disorders on this disease.

## Data Availability Statement

All datasets generated for this study are included in the article/Supplementary Material.

## Ethics Statement

The studies involving human participants were reviewed and approved by Institutional Review Boards and Ethics Committees of Hospital of Chengdu University of Traditional Chinese Medicine (Approved number: 2018KL-064). The patients/participants provided their written informed consent to participate in this study.

## Author Contributions

PZ and DC were responsible for this study. PZ, TY, QL, and ZM contributed to the study conception and design. QL and ZL participated in material preparation and patient recruitment. ZM, FR, GL, and XH contributed to patient recruitment. TY, RS, and QL performed the data collection and data analysis. TY wrote the first draft of the manuscript. All authors commented on previous versions of the manuscript, read, and approved the final manuscript.

## Conflict of Interest

The authors declare that the research was conducted in the absence of any commercial or financial relationships that could be construed as a potential conflict of interest.

## References

[1] Najari BB Kashanian JA. Erectile dysfunction. JAMA. (2016) 316:1838. 10.1001/jama.2016.12284 27802547

[2] Rosen RC Fisher WA Eardley I Niederberger C Nadel A Sand M The multinational Men's Attitudes to Life Events and Sexuality. (MALES) study: I. Prevalence of erectile dysfunction and related health concerns in the general population. Curr Med Res Opin. (2004) 20:607–17. 10.1185/030079904125003467 15171225

[3] Quilter M Hodges L von Hurst P Borman B Coad J. Male sexual function in New Zealand: a population-based cross-sectional survey of the prevalence of erectile dysfunction in men aged 40-70 years. J Sex Med. (2017) 14:928–36. 10.1016/j.jsxm.2017.05.011 28673435

[4] Nguyen HMT Gabrielson AT Hellstrom WJG. Erectile dysfunction in young men-a review of the prevalence and risk factors. Sex Med Rev. (2017) 5:508–20. 10.1016/j.sxmr.2017.05.004 28642047

[5] Boddi V Corona G Fisher AD Mannucci E Ricca V Sforza A. “It takes two to tango”: the relational domain in a cohort of subjects with erectile dysfunction. (ED). J Sex Med. (2012) 9:3126–36. 10.1111/j.1743-6109.2012.02948.x 23036015

[6] Li HJ Bai WJ Dai YT Xu WP Wang CN Li HZ. An analysis of treatment preferences and sexual quality of life outcomes in female partners of Chinese men with erectile dysfunction. Asian J Androl. (2016) 18:773–9. 10.4103/1008-682X.159719 26459780 PMC5000803

[7] Yafi FA Jenkins L Albersen M Corona G Isidori AM Goldfarb S. Erectile dysfunction. Nat Rev Dis Primers. (2016) 2:16003. 10.1038/nrdp.2016.3 27188339 PMC5027992

[8] Coward RM Stetter C Kunselman A Trussell JC Lindgren MC Alvero RR. Fertility related quality of life, gonadal function and erectile dysfunction in male partners of couples with unexplained infertility. J Urol. (2019) 202:379–84. 10.1097/JU.0000000000000205 30835629 PMC6686175

[9] Shamloul R Ghanem H. Erectile dysfunction. Lancet. (2013) 381:153–65. 10.1016/S0140-6736(12)60520-0 23040455

[10] McMahon CG. Current diagnosis and management of erectile dysfunction. Med J Aust. (2019) 210:469–76. 10.5694/mja2.50167 31099420

[11] Rastrelli G Maggi M Erectile dysfunction in fit and healthy young men: psychological or pathological? Transl Androl Urol. (2017) 6:79–90. 10.21037/tau.2016.09.06 28217453 PMC5313296

[12] Chen L Shi GR Huang DD Li Y Ma CC Shi M. Male sexual dysfunction: a review of literature on its pathological mechanisms, potential risk factors, and herbal drug intervention. Biomed Pharmacother. (2019) 112:108585. 10.1016/j.biopha.2019.01.046 30798136

[13] Prieto D. Physiological regulation of penile arteries and veins. Int J Impot Res. (2008) 20:17–29. 10.1038/sj.ijir.3901581 17637789

[14] Brunetti M Babiloni C Ferretti A Del Gratta C Merla A Olivetti Belardinelli M. Hypothalamus, sexual arousal and psychosexual identity in human males: a functional magnetic resonance imaging study. Eur J Neurosci. (2008) 27:2922–7. 10.1111/j.1460-9568.2008.06241.x 18588532

[15] Courtois F Carrier S Charvier K Guertin PA Journel NM. The control of male sexual responses. Curr Pharm Des. (2013) 19:4341–56. 10.2174/13816128113199990333 23360268

[16] Wu SL Chow MSM L JY Yang J Zhou H Yew DT. Visual sexual stimulation and erection, a brief review with new fMRI data. Curr Med Chem. (2017) 24:1139–46. 10.2174/0929867323666161213102528 27978801

[17] Cera N Delli Pizzi S Di Pierro ED Gambi F Tartaro A Vicentini C. Macrostructural alterations of subcortical grey matter in psychogenic erectile dysfunction. PLoS ONE. (2012) 7:e39118. 10.1371/journal.pone.0039118 22723943 PMC3377616

[18] Cera N Di Pierro ED Ferretti A Tartaro A Romani GL Perrucci MG. Brain networks during free viewing of complex erotic movie: new insights on psychogenic erectile dysfunction. PLoS ONE. (2014) 9:e105336. 10.1371/journal.pone.0105336 25126947 PMC4134311

[19] Zhao L Guan M Zhang X Karama S Khundrakpam B Wang M. Structural insights into aberrant cortical morphometry and network organization in psychogenic erectile dysfunction. Hum Brain Mapp. (2015) 36:4469–82. 10.1002/hbm.22925 26264575 PMC6869733

[20] Wang Y Dong M Guan M Wu J He Z Zou Z. Aberrant insula-centered functional connectivity in psychogenic erectile dysfunction patients: a resting-state fMRI study. Front Hum Neurosci. (2017) 11:221. 10.3389/fnhum.2017.00221 28559803 PMC5433384

[21] Chen J Chen Y Gao Q Chen G Dai Y Yao Z. Impaired prefrontal-amygdala pathway, self-reported emotion, and erection in psychogenic erectile dysfunction patients with normal nocturnal erection. Front Hum Neurosci. (2018) 12:157. 10.3389/fnhum.2018.00157 29740301 PMC5928255

[22] Chen J Huang X Liu S Lu C Dai Y Yao Z. Disrupted topological properties of brain networks in erectile dysfunction patients owing predominantly to psychological factors: a structural and functional neuroimaging study. Andrology. (2020) 8:381–91. 10.1111/andr.12684 31468742

[23] Xu Z Yang X Gao M Liu L Sun J Liu P. Abnormal resting-state functional connectivity in the whole brain in lifelong premature ejaculation patients based on machine learning approach. Front Neurosci. (2019) 13:448. 10.3389/fnins.2019.00448 31139043 PMC6519512

[24] Chen J Huang X Lu C Liu T Dai Y Yao Z Graph analysis of DTI-based connectome: decreased local efficiency of subcortical regions in PE patients with high sympathetic activity. Andrology. (2020) 8:400–6. 10.1111/andr.12701 31532583

[25] Chen J Yang J Huang X Ni L Fan Q Liu T. Reduced segregation and integration of structural brain network associated with sympathetic and dorsal penile nerve activity in anejaculation patients: a graph-based connectome study. Andrology. (2020) 8:392–9. 10.1111/andr.12715 31610095

[26] Zhang P Liu J Li G Pan J Li Z Liu Q. White matter microstructural changes in psychogenic erectile dysfunction patients. Andrology. (2014) 2:379–85. 10.1111/j.2047-2927.2014.00191.x 24711250

[27] Zang YF He Y Zhu CZ Cao QJ Sui MQ Liang M. Altered baseline brain activity in children with ADHD revealed by resting-state functional MRI. Brain Dev. (2007) 29:83–91. 10.1016/j.braindev.2006.07.002 16919409

[28] Smitha KA Akhil Raja K Arun KM Rajesh PG Thomas B Kapilamoorthy TR. Resting state fMRI: a review on methods in resting state connectivity analysis and resting state networks. Neuroradiol J. (2017) 30:305–17. 10.1177/1971400917697342 28353416 PMC5524274

[29] Cheng R Qi H Liu Y Zhao S Li C Liu C. Abnormal amplitude of low-frequency fluctuations and functional connectivity of resting-state functional magnetic resonance imaging in patients with leukoaraiosis. Brain Behav. (2017) 7:e00714. 10.1002/brb3.714 28638719 PMC5474717

[30] Tang Y Zhou Q Chang M Chekroud A Gueorguieva R Jiang X. Altered functional connectivity and low-frequency signal fluctuations in early psychosis and genetic high risk. Schizophr Res. (2019) 210:172–9. 10.1016/j.schres.2018.12.041 30685394

[31] Wang XF Zhu JC Deng CH Guidelines for the Diagnosis and Treatment of Chinese Male Diseases. 2013 ed. Beijing: People's Medical Publishing House (2013).

[32] Zung WWK. A rating instrument for anxiety disorders. Psychosomatics. (1971) 12:371–9. 10.1016/S0033-3182(71)71479-0 5172928

[33] Zung WW. A self-rating depression scale. Arch Gen Psychiatry. (1965) 12:63–70. 10.1001/archpsyc.1965.01720310065008 14221692

[34] Rosen RC Cappelleri JC Smith MD Lipsky J Pena BM. Development and evaluation of an abridged, 5-item version of the International Index of Erectile Function. (IIEF-5) as a diagnostic tool for erectile dysfunction. Int J Impot Res. (1999) 11:319–26. 10.1038/sj.ijir.3900472 10637462

[35] Cappelleri JC Rosen RC. The Sexual Health Inventory for Men. (SHIM): a 5-year review of research and clinical experience. Int J Impot Res. (2005) 17:307–19. 10.1038/sj.ijir.3901327 15875061

[36] Althof SE Cappelleri JC Shpilsky A Stecher V Diuguid C Sweeney M. Treatment responsiveness of the Self-Esteem and Relationship questionnaire in erectile dysfunction. Urology. (2003) 61:888–92. 10.1016/S0090-4295(03)00041-4 12735997

[37] Cappelleri JC Althof SE Siegel RL Shpilsky A Bell SS Duttagupta S. Development and validation of the Self-Esteem And Relationship. (SEAR) questionnaire in erectile dysfunction. Int J Impot Res. (2004) 16:30–8. 10.1038/sj.ijir.3901095 14963468

[38] McCabe MP Althof SE. A systematic review of the psychosocial outcomes associated with erectile dysfunction: does the impact of erectile dysfunction extend beyond a man's inability to have sex? J Sex Med. (2014) 11:347–63. 10.1111/jsm.12374 24251371

[39] Porst H Gilbert C Collins S Huang X Symonds T Stecher V. Development and validation of the quality of erection questionnaire. J Sex Med. (2007) 4:372–81. 10.1111/j.1743-6109.2006.00422.x 17367432

[40] Yan CG Wang XD Zuo XN Zang YF. DPABI: Data Processing and Analysis for (Resting-State) brain imaging. Neuroinformatics. (2016) 14:339–51. 10.1007/s12021-016-9299-4 27075850

[41] Power JD Barnes KA Snyder AZ Schlaggar BL Petersen SE. Spurious but systematic correlations in functional connectivity MRI networks arise from subject motion. Neuroimage. (2012) 59:2142–54. 10.1016/j.neuroimage.2011.10.018 22019881 PMC3254728

[42] Fiorenzato E Strafella AP Kim J Schifano R Weis L Antonini A. Dynamic functional connectivity changes associated with dementia in Parkinson's disease. Brain. (2019) 142:2860–72. 10.1093/brain/awz192 31280293 PMC6736370

[43] Friston KJ Williams S Howard R Frackowiak RS Turner R. Movement-related effects in fMRI time-series. Magn Reson Med. (1996) 35:346–55. 10.1002/mrm.1910350312 8699946

[44] Yan CG Cheung B Kelly C Colcombe S Craddock RC Di Martino A. A comprehensive assessment of regional variation in the impact of head micromovements on functional connectomics. Neuroimage. (2013) 76:183–201. 10.1016/j.neuroimage.2013.03.004 23499792 PMC3896129

[45] Biswal B Yetkin FZ Haughton VM Hyde JS. Functional connectivity in the motor cortex of resting human brain using echo-planar MRI. Magn Reson Med. (1995) 34:537–41. 10.1002/mrm.1910340409 8524021

[46] Chumbley JR Friston KJ. False discovery rate revisited: FDR and topological inference using Gaussian random fields. Neuroimage. (2009) 44:62–70. 10.1016/j.neuroimage.2008.05.021 18603449

[47] Chumbley J Worsley K Flandin G Friston K. Topological FDR for neuroimaging. Neuroimage. (2010) 49:3057–64. 10.1016/j.neuroimage.2009.10.090 19944173 PMC3221040

[48] Zeng F Sun R He Z Chen Y Lei D Yin T. Altered functional connectivity of the amygdala and sex differences in functional dyspepsia. Clin Transl Gastroenterol. (2019) 10:e00046. 10.14309/ctg.0000000000000046 31136362 PMC6613861

[49] Preacher KJ Hayes AF. Asymptotic and resampling strategies for assessing and comparing indirect effects in multiple mediator models. Behav Res Methods. (2008) 40:879–91. 10.3758/BRM.40.3.879 18697684

[50] Gu Y Vorburger RS Gazes Y Habeck CG Stern Y Luchsinger JA. White matter integrity as a mediator in the relationship between dietary nutrients and cognition in the elderly. Ann Neurol. (2016) 79:1014–25. 10.1002/ana.24674 27129740 PMC4884180

[51] Callaghan BL Dandash O Simmons JG Schwartz O Byrne ML Sheeber L. Amygdala resting connectivity mediates association between maternal aggression and adolescent major depression: a 7-year longitudinal study. J Am Acad Child Adolesc Psychiatry. (2017) 56:983–91.e983. 10.1016/j.jaac.2017.09.415 29096781

[52] Tiefer L Schuetz-Mueller D. Psychological issues in diagnosis and treatment of erectile disorders. Urol Clin North Am. (1995) 22:767–73. 7483127

[53] Cappelleri JC Bell SS Althof SE Siegel RL Stecher VJ. Comparison between sildenafil-treated subjects with erectile dysfunction and control subjects on the Self-Esteem And Relationship questionnaire. J Sex Med. (2006) 3:274–82. 10.1111/j.1743-6109.2005.00205.x 16490020

[54] Moncada I Martinez-Jabaloyas JM Rodriguez-Vela L Gutierrez PR Giuliano F Koskimaki J. Emotional changes in men treated with sildenafil citrate for erectile dysfunction: a double-blind, placebo-controlled clinical trial. J Sex Med. (2009) 6:3469–77. 10.1111/j.1743-6109.2009.01514.x 19796051

[55] Patel HR Ilo D Shah N Cuzin B Chadwick D Andrianne R. Effects of tadalafil treatment after bilateral nerve-sparing radical prostatectomy: quality of life, psychosocial outcomes, and treatment satisfaction results from a randomized, placebo-controlled phase IV study. BMC Urol. (2015) 15:31. 10.1186/s12894-015-0022-9 25879460 PMC4419565

[56] Tang WH Zhuang XJ Ma LL Hong K Zhao LM Liu DF Effect of sildenafil on erectile dysfunction and improvement in the quality of sexual life in China: a multi-center study. Int J Clin Exp Med. (2015) 8:11539–43. 26379977 PMC4565360

[57] Ponomarenko GN Bin'iash TG Raigorodskii Iu M Guliaev AS Shul'diakov VA Kiriliuk AM. Transcranial magneto- and electrostimulation in patients with obesity and erectile dysfunction [Article in Russian]. Vopr Kurortol Fizioter Lech Fiz Kult. (2009) 5:30–3. 19886019

[58] Andersson E Walén C Hallberg J Paxling B Dahlin M Almlöv J. A randomized controlled trial of guided Internet-delivered cognitive behavioral therapy for erectile dysfunction. J Sex Med. (2011) 8:2800–9. 10.1111/j.1743-6109.2011.02391.x 21797983

[59] Wassersug R Wibowo E. Non-pharmacological and non-surgical strategies to promote sexual recovery for men with erectile dysfunction. Transl Androl Urol. (2017) 6(Suppl. 5):S776–94. 10.21037/tau.2017.04.09 29238658 PMC5715194

[60] Spinella M. The role of prefrontal systems in sexual behavior. Int J Neurosci. (2007) 117:369–85. 10.1080/00207450600588980 17365121

[61] Walter M Witzel J Wiebking C Gubka U Rotte M Schiltz K. Pedophilia is linked to reduced activation in hypothalamus and lateral prefrontal cortex during visual erotic stimulation. Biol Psychiatry. (2007) 62:698–701. 10.1016/j.biopsych.2006.10.018 17400196

[62] Cheng JC Secondary J Burke WH Fedoroff JP Dwyer RG. Neuroimaging and sexual behavior: identification of regional and functional differences. Curr Psychiatry Rep. (2015) 17:55. 10.1007/s11920-015-0593-x 25980508

[63] Leon-Carrion J Martin-Rodriguez JF Damas-Lopez J Pourrezai K Izzetoglu K Barroso YMJM. Does dorsolateral prefrontal cortex. (DLPFC) activation return to baseline when sexual stimuli cease? The role of DLPFC in visual sexual stimulation. Neurosci Lett. (2007) 416:55–60. 10.1016/j.neulet.2007.01.058 17316990

[64] Seok JW Sohn JH. Neural substrates of sexual desire in individuals with problematic hypersexual behavior. Front Behav Neurosci. (2015) 9:321. 10.3389/fnbeh.2015.00321 26648855 PMC4663274

[65] Cacioppo S. Neuroimaging of female sexual desire and hypoactive sexual desire disorder. Sex Med Rev. (2017) 5:434–44. 10.1016/j.sxmr.2017.07.006 28865901

[66] Seeley WW Menon V Schatzberg AF Keller J Glover GH Kenna H. Dissociable intrinsic connectivity networks for salience processing and executive control. J Neurosci. (2007) 27:2349–56. 10.1523/JNEUROSCI.5587-06.2007 17329432 PMC2680293

[67] Hoffmann M. The human frontal lobes and frontal network systems: an evolutionary, clinical, and treatment perspective. ISRN Neurol. (2013) 2013:892459. 10.1155/2013/892459 23577266 PMC3612492

[68] Beauregard M Levesque J Bourgouin P. Neural correlates of conscious self-regulation of emotion. J Neurosci. (2001) 21:Rc165. 10.1523/JNEUROSCI.21-18-j0001.2001 11549754 PMC6763007

[69] Laird AR Eickhoff SB Li K Robin DA Glahn DC Fox PT. Investigating the functional heterogeneity of the default mode network using coordinate-based meta-analytic modeling. J Neurosci. (2009) 29:14496–505. 10.1523/JNEUROSCI.4004-09.2009 19923283 PMC2820256

[70] Andrews-Hanna JR Smallwood J Spreng RN. The default network and self-generated thought: component processes, dynamic control, and clinical relevance. Ann N Y Acad Sci. (2014) 1316:29–52. 10.1111/nyas.12360 24502540 PMC4039623

[71] Hu SH Wei N Wang QD Yan LQ Wei EQ Zhang MM. Patterns of brain activation during visually evoked sexual arousal differ between homosexual and heterosexual men. Am J Neuroradiol. (2008) 29:1890–6. 10.3174/ajnr.A1260 18768725 PMC8118934

[72] Huh J Park K Hwang IS Jung SI Kim HJ Chung TW . Brain activation areas of sexual arousal with olfactory stimulation in men: a preliminary study using functional MRI. J Sex Med. (2008) 5:619–25. 10.1111/j.1743-6109.2007.00717.x 18221282

[73] Stoleru S Fonteille V Cornelis C Joyal C Moulier V. Functional neuroimaging studies of sexual arousal and orgasm in healthy men and women: a review and meta-analysis. Neurosci Biobehav Rev. (2012) 36:1481–509. 10.1016/j.neubiorev.2012.03.006 22465619

[74] Sumich AL Kumari V Sharma T. Neuroimaging of sexual arousal: research and clinical utility. Hosp Med. (2003) 64:28–33. 10.12968/hosp.2003.64.1.2378 12572332

[75] Poeppl TB Langguth B Laird AR Eickhoff SB. Meta-analytic evidence for neural dysactivity underlying sexual dysfunction. J Sex Med. (2019) 16:614–17. 10.1016/j.jsxm.2019.02.012 30926513 PMC7211028

[76] Buckner RL Andrews-Hanna JR Schacter DL. The brain's default network: anatomy, function, and relevance to disease. Ann N Y Acad Sci. (2008) 1124:1–38. 10.1196/annals.1440.011 18400922

[77] Raichle ME. The brain's default mode network. Annu Rev Neurosci. (2015) 38:433–47. 10.1146/annurev-neuro-071013-014030 25938726

[78] Pan W Liu C Yang Q Gu Y Yin S Chen A. The neural basis of trait self-esteem revealed by the amplitude of low-frequency fluctuations and resting state functional connectivity. Soc Cogn Affect Neurosci. (2016) 11:367–76. 10.1093/scan/nsv119 26400859 PMC4769622

[79] Fransson P Marrelec G. The precuneus/posterior cingulate cortex plays a pivotal role in the default mode network: evidence from a partial correlation network analysis. Neuroimage. (2008) 42:1178–84. 10.1016/j.neuroimage.2008.05.059 18598773

[80] Hoefler A Athenstaedt U Corcoran K Ebner F Ischebeck A. Coping with self-threat and the evaluation of self-related traits: an fMRI study. PLoS ONE. (2015) 10:e0136027. 10.1371/journal.pone.0136027 26333130 PMC4558049

[81] Seghier ML. The angular gyrus: multiple functions and multiple subdivisions. Neuroscientist. (2013) 19:43–61. 10.1177/1073858412440596 22547530 PMC4107834

[82] Thakral PP Madore KP Schacter DL. A role for the left angular gyrus in episodic simulation and memory. J Neurosci. (2017) 37:8142–9. 10.1523/JNEUROSCI.1319-17.2017 28733357 PMC5566865

[83] Ferretti A Caulo M Del Gratta C Di Matteo R Merla A Montorsi F. Dynamics of male sexual arousal: distinct components of brain activation revealed by fMRI. Neuroimage. (2005) 26:1086–96. 10.1016/j.neuroimage.2005.03.025 15961048

[84] Seok JW Park MS Sohn JH. Neural pathways in processing of sexual arousal: a dynamic causal modeling study. Int J Impot Res. (2016) 28:184–8. 10.1038/ijir.2016.27 27278664

[85] Carson CC Dean JD Erectile dysfunction — etiology and risk factors. Management of Erectile Dysfunction in Clinical Practice. London: Springer London (2007). p. 19–39.

[86] Grandjean J Azzinnari D Seuwen A Sigrist H Seifritz E Pryce CR. Chronic psychosocial stress in mice leads to changes in brain functional connectivity and metabolite levels comparable to human depression. Neuroimage. (2016) 142:544–52. 10.1016/j.neuroimage.2016.08.013 27520750

[87] Wise T Marwood L Perkins AM Herane-Vives A Joules R Lythgoe DJ. Instability of default mode network connectivity in major depression: a two-sample confirmation study. Transl Psychiatry. (2017) 7:e1105. 10.1038/tp.2017.40 28440813 PMC5416685

[88] Akiki TJ Averill CL Wrocklage KM Scott JC Averill LA Schweinsburg B. Default mode network abnormalities in posttraumatic stress disorder: a novel network-restricted topology approach. Neuroimage. (2018) 176:489–98. 10.1016/j.neuroimage.2018.05.005 29730491 PMC5976548

[89] Lee D Lee J Lee JE Jung YC. Altered functional connectivity in default mode network in Internet gaming disorder: influence of childhood ADHD. Prog Neuropsychopharmacol Biol Psychiatry. (2017) 75:135–41. 10.1016/j.pnpbp.2017.02.005 28174127

[90] Uytun MC Karakaya E Oztop DB Gengec S Gumus K Ozmen S. Default mode network activity and neuropsychological profile in male children and adolescents with attention deficit hyperactivity disorder and conduct disorder. Brain Imaging Behav. (2017) 11:1561–70. 10.1007/s11682-016-9614-6 27738997

[91] Li R Zhang S Yin S Ren W He R Li J. The fronto-insular cortex causally mediates the default-mode and central-executive networks to contribute to individual cognitive performance in healthy elderly. Hum Brain Mapp. (2018) 39:4302–11. 10.1002/hbm.24247 29974584 PMC6866622

[92] Bressler SL Menon V. Large-scale brain networks in cognition: emerging methods and principles. Trends Cogn Sci. (2010) 14:277–90. 10.1016/j.tics.2010.04.004 20493761

[93] Mulders PC van Eijndhoven PF Schene AH Beckmann CF Tendolkar I. Resting-state functional connectivity in major depressive disorder: a review. Neurosci Biobehav Rev. (2015) 56:330–44. 10.1016/j.neubiorev.2015.07.014 26234819

[94] Akiki TJ Averill CL Abdallah CG. A network-based neurobiological model of PTSD: evidence from structural and functional neuroimaging studies. Curr Psychiatry Rep. (2017) 19:81. 10.1007/s11920-017-0840-4 28924828 PMC5960989

[95] van Oort J Tendolkar I Hermans EJ Mulders PC Beckmann CF Schene AH. How the brain connects in response to acute stress: a review at the human brain systems level. Neurosci Biobehav Rev. (2017) 83:281–97. 10.1016/j.neubiorev.2017.10.015 29074385

