# Supplementary material for: Associations Between Altered Cerebral Activity Patterns and Psychosocial Disorders in Patients With Psychogenic Erectile Dysfunction: A Mediation Analysis of fMRI
Source: Front Psychiatry. 2020 Oct 27;11:583619. doi: 10.3389/fpsyt.2020.583619 (PMC7652753; doi:10.3389/fpsyt.2020.583619)
Supplement: Supplementary file 1 [file Data_Sheet_1.PDF]

Suppl. 1

Given the significant correlation between the duration and the SEARQ score (*r*= -0.513, *p*= 0.003), we conducted the correlation analyses between the neuroimaging data and clinical symptoms with duration as an additional covariate to exclude its impact. The results demonstrated that the ALFF of left dlPFC was positively correlated with the IIEF-5 score (r= 0.548, p= 0.0014) and SEARQ score (r= 0.509, p= 0.0034), the ROI-left PCC & precuneus connectivity was positively correlated with IIEF-5 score (r= 0.565, p= 0.0009) and SEARQ score (r= 0.616, p= 0.0003), and the ROI-left AG connectivity was positively correlated with IIEF-5 score (r= 0.444, p= 0.0122) and SEARQ score (r= 0.391, p= 0.0295) with age, BMI, mean FD, and duration as covariates (Fig. S1).

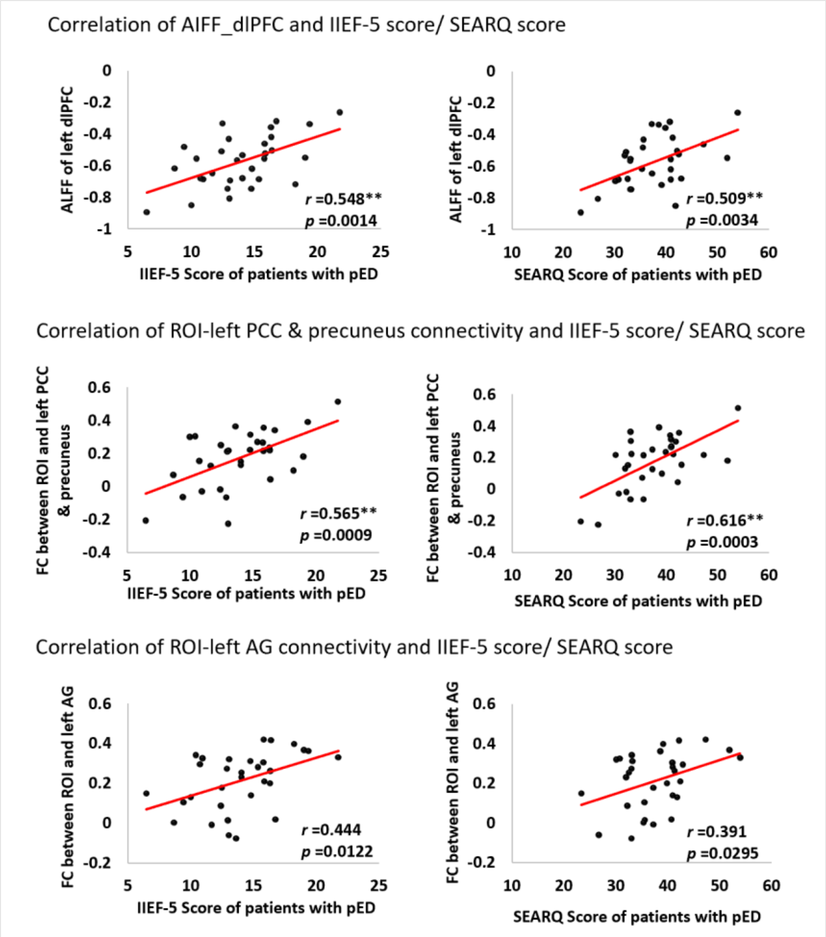

Fig. S1 The scatter plots of correlation analyses between neuroimaging data and clinical symptoms with age, BMI, mean FD, and duration as covariates. ** indicates $p < 0.05$ with Bonferroni corrected.
